# The structure, functional evolution, and evolutionary trajectories of the H^+^-PPase gene family in plants

**DOI:** 10.1186/s12864-020-6604-2

**Published:** 2020-03-02

**Authors:** Yiming Zhang, Xue Feng, Lihui Wang, Yanping Su, Zhuodong Chu, Yanxiang Sun

**Affiliations:** 1 College of Life Sciences, Langfang Normal University, Langfang, 065000 China; 2 College of Plant Protection, Fujian Agricultural and Forestry University, Fuzhou, 350000 China

**Keywords:** H^+^-PPase gene family, Duplication events, Functional divergence, Positive selection, Evolutionary trajectories

## Abstract

**Background:**

The H^+^-PPase (pyrophosphatase) gene family is an important class of proton transporters that play key roles in plant development and stress resistance. Although the physiological and biochemical functions of H^+^-PPases are well characterized, the structural evolution and functional differentiation of this gene family remain unclear.

**Results:**

We identified 124 H^+^-PPase members from 27 plant species using complete genomic data obtained from algae to angiosperms. We found that all analyzed plants carried H^+^-PPase genes, and members were not limited to the two main types (type I and II). Differentiation of this gene family occurred early in evolutionary history, probably prior to the emergence of algae. The type I and II H^+^-PPase genes were retained during the subsequent evolution of higher plants, and their copy numbers increased rapidly in some angiosperms following whole-genome duplication (WGD) events, with obvious expression pattern differentiation among the new copies. We found significant functional divergence between type I and II H^+^-PPase genes, with both showing evidence for positive selection pressure. We classified angiosperm type I H^+^-PPases into subtypes Ia and non-Ia, which probably differentiated at an early stage of angiosperm evolution. Compared with non-Ia subtype, the Ia subtype appears to confer some advantage in angiosperms, as it is highly conserved and abundantly expressed, but shows no evidence for positive selection.

**Conclusions:**

We hypothesized that there were many types of H^+^-PPase genes in the plant ancestral genome, and that different plant groups retained different types of these genes. In the early stages of angiosperm evolution, the type I H^+^-PPase genes differentiated into various subtypes. In addition, the expression pattern varied not only among genes of different types or subtypes, but also among copies of the same subtype. Based on the expression patterns and copy numbers of H^+^-PPase genes in higher plants, we propose two possible evolutionary trajectories for this gene family.

## Background

H^+^-PPases (proton-translocating pyrophosphatase) are a branch of membrane-bound pyrophosphatase enzymes that hydrolyze inorganic pyrophosphate (PPi) to obtain energy and transport protons across the cell membrane [1]. These enzymes were considered to be unique to plants and photosynthetic bacteria, but recent studies have found that this gene family is also widespread among bacteria, archaea, and primitive parasites, and emerged in the Last Universal Common Ancestor (LUCA) [2].

The H^+^-PPase proteins can generally be divided into two types according to their demand for potassium (K^+^) ions: type I reaches its peak activity in the presence of K^+^, while type II does not depend on the presence of K^+^ ions [3]. The dependence of type I H^+^-PPase proteins on K^+^ ions is determined by the GNxxAAIG motif, in which the first alanine (A) and the last glycine (G) residues play an important functional role [4, 5].

Most research on plant H^+^-PPases has focused on the type I H^+^-PPases, which are located on the vacuolar membrane and are thus known as vacuolar proton pyrophosphatases (V-PPase); e.g., *Arabidopsis* AVP1 (At1g15690) [6–8]. In plant cells, type I H^+^-PPases obtain energy through the hydrolysis of PPi to transport protons across the vacuole membrane, and adjust the pH in the vacuole and cytoplasm [9]. Type I proteins are widely involved in metabolic processes such as the enrichment of metal ions in the vacuole [10] and hormone and nutrient transfer [9]. Overexpression of type I H^+^-PPase genes can significantly enhance the ability of plants to cope with abiotic stresses, such as anoxia or chilling [11], lack of nutrition [12], drought, and high salt levels [13, 14]. This can also promote plant vegetative growth and produce plants with large biomass [9, 13]. There are differences in the copy number of H^+^-PPase genes in different plants. Different members of this gene family may have specific expression in different tissues, organs, or during different developmental stages, but there is currently no compelling evidence to support this [10]. Both type I and type II H^+^-PPases have the same active site but have significant differences in subcellular localization and expression levels. For example, *Arabidopsis* AVP2 (At1g78920, a type II H^+^-PPase) is located in the Golgi apparatus, and its expression level is much lower than that of type I H^+^-PPases [15].

The structural evolution and functional differentiation of this gene family have not been reported systematically. With the availability of increasing numbers of plant genomes and the continuous improvement of the available protein tertiary structure model [16, 17], we currently have the ability to study the H^+^-PPase gene family from a wider perspective. In the present study, we selected 27 plant species with different taxonomic relationships to identify and study the structure of H^+^-PPase gene family members at the whole genome level. The evolutionary relationships and expression patterns of different members of this gene family were investigated. Further we performed functional diversity analysis and positive selection analysis to explore the evolution of their structure and function. Based on the research results, we provide a theoretical basis for further research on the function of H^+^-PPase genes in plants.

## Results

### Cross-species distribution of H^+^-PPase genes in plants

Twenty-seven plants with relatively complete genome annotations were selected for the identification of H^+^-PPase gene family members. HMMER v 3.1 [18] was used to search for candidate genes in complete protein sequence data of different species (hidden Markov model number: PF03030). After identification and filtering, 124 H^+^-PPase gene family members were identified (Table 1, Additional file 1). All plant species evaluated in the present study contained at least one member of the H^+^-PPase gene family. No algae contained more than three of these genes, and many contained only one H^+^-PPase gene (e.g., *Cyanidioschyzon merolae*, *Dunaliella salina*, *Chlamydomonas reinhardtii*, *Volvox carteri*) (Table 1, Additional file 1). In contrast, the angiosperm species had several H^+^-PPase genes, with the eudicot upland cotton (*Gossypium hirsutum*), which reunited the A- and D-genomes in recent history [19], having as many as 16 H^+^-PPase genes. In the monocots, with ten genes, maize (*Zea mays*) had second highest number of H^+^-PPase genes. With just two H^+^-PPase genes, the magnoliid columbine (*Aquilegia caerulea*) was the angiosperm with the fewest H^+^-PPase genes. We observed that the expansion of the H^+^-PPase gene family was concentrated in the angiosperms.

**Table 1:** Characterization of the members of the H^+^-PPase gene family in 27 plant species

Species groups	Species	Number of members	Annotation gene names
Red algae	*Cyanidioschyzon merolae*	1	CMO102C
	*Galdieria sulphuraria*	3	Gasu_15740, Gasu_15900, Gasu_28190
Green algae	*Micromonas pusilla*	3	MicpuC2.estExt_fgenesh1_pm.C_20025, MicpuC2.estExt_fgenesh1_pg.C_30365, MicpuC2.estExt_Genewise1Plus.C_60613
	*Ostreococcus lucimarinus*	3	eugene.0400010383, estExt_Genewise_ext.C_Chr_10614, e_gwEuk.1.151.1
	*Dunaliella salina*	1	Dusal.0221 s00015
	*Chlamydomonas reinhardtii*	1	CHLRE_09g394436v5
	*Volvox carteri*	1	Vocar.0009 s0186
Bryophytes	*Physcomitrella patens*	4	**PHYPA_000091, PHYPA_000092,** PHYPA_001647, PHYPA_021933
Ferns	*Selaginella moellendorffii*	4	SELMODRAFT_157618, SELMODRAFT_156843, SELMODRAFT_270614, SELMODRAFT_270204
Angiosperms	*Amborella trichopoda*	3	AMTR_s00025p00194920, AMTR_s00003p00014700, AMTR_s00033p00195690
	*Spirodela polyrhiza*	3	Spipo1G0016600, Spipo31G0011200, Spipo3G0025000
	*Musa acuminata*	8	GSMUA_Achr1G26020, GSMUA_Achr2G05200, GSMUA_Achr6G36430, GSMUA_Achr7G20850, GSMUA_Achr3G13280, GSMUA_Achr5G23480, GSMUA_Achr5G13160, GSMUA_Achr8G06450
	*Triticum aestivum*	8	Traes_6BL_E905C1C95, Traes_6AL_5F50463BE, Traes_6DL_FC95036E1, Traes_1DS_EF07A3CBD, Traes_1BS_1514DE4E9, Traes_7DL_3BA7EF708, Traes_7BS_55CB27B54, Traes_7AL_AA1B5DFB5
	*Zea mays*	10	Zm00008a030532, Zm00008a018655, Zm00008a012212, Zm00008a033578, Zm00008a025249, Zm00008a034646, Zm00008a011941, Zm00008a002892, Zm00008a025306, Zm00008a021157
	*Aquilegia coerulea*	2	Aqcoe7G285200, Aqcoe7G376400
	*Beta vulgaris*	3	BVRB_8g193170, BVRB_9g219460, BVRB_7g177860
	*Helianthus annuus*	6	HannXRQ_Chr10g0314301, HannXRQ_Chr12g0357171, HannXRQ_Chr16g0500291, HannXRQ_Chr04g0098051, HannXRQ_Chr05g0129041, HannXRQ_Chr09g0242721
	*Solanum lycopersicum*	6	Solyc04g071880.2, Solyc07g007600.2, Solyc03g117480.2, Solyc12g009840.1, Solyc01g100390.2, Solyc06g068240.2
	*Kalanchoe fedtschenkoi*	6	Kaladp0048s0603, Kaladp0011s0323, Kaladp0036s0139, Kaladp0037s0358, Kaladp0048s0764, Kaladp0095s0302
	*Vitis vinifera*	4	VIT_09s0002g07880, VIT_09s0054g00700, VIT_14s0060g01280, VIT_11s0118g00350
	*Arabidopsis thaliana*	3	AT1G15690, AT1G16780, AT1G78920
	*Theobroma cacao*	4	TCM_026755, TCM_027289, TCM_027736, TCM_038184
	*Gossypium hirsutum*	16	Gohir.D06G120900, Gohir.A06G116100, Gohir.A10G001500, Gohir.A05G122300, Gohir.D05G123200, Gohir.A05G013400, Gohir.A06G052500, Gohir.D06G051500, Gohir.A13G201700, Gohir.A09G085900, Gohir.D08G100800, Gohir.D09G086000, Gohir.D13G207500, Gohir.A08G089700, Gohir.D10G001600, Gohir.A13G112000
	*Populus trichocarpa*	6	Potri.010G254200, Potri.018G122700, Potri.013G009400, Potri.006G063000, Potri.005G018700, Potri.018G119500
	*Cucumis sativus*	3	Csa_1G212840, Csa_2G033950, Csa_7G447180
	*Glycine max*	8	GLYMA_08G225500, GLYMA_08G214300, GLYMA_20G098300, GLYMA_13G162800, GLYMA_17G108500, GLYMA_07G028500, GLYMA_07G001500, GLYMA_10G147500
	*Prunus persica*	4	PRUPE_6G313800, PRUPE_3G091900, PRUPE_3G024800, PRUPE_7G250800

Notes: The two genes highlighted by the bold typeface indicate that the pair of genes are tandem repeats

### Phylogenetic analysis of the plant H^+^-PPase gene family members

**Fig. 1:**
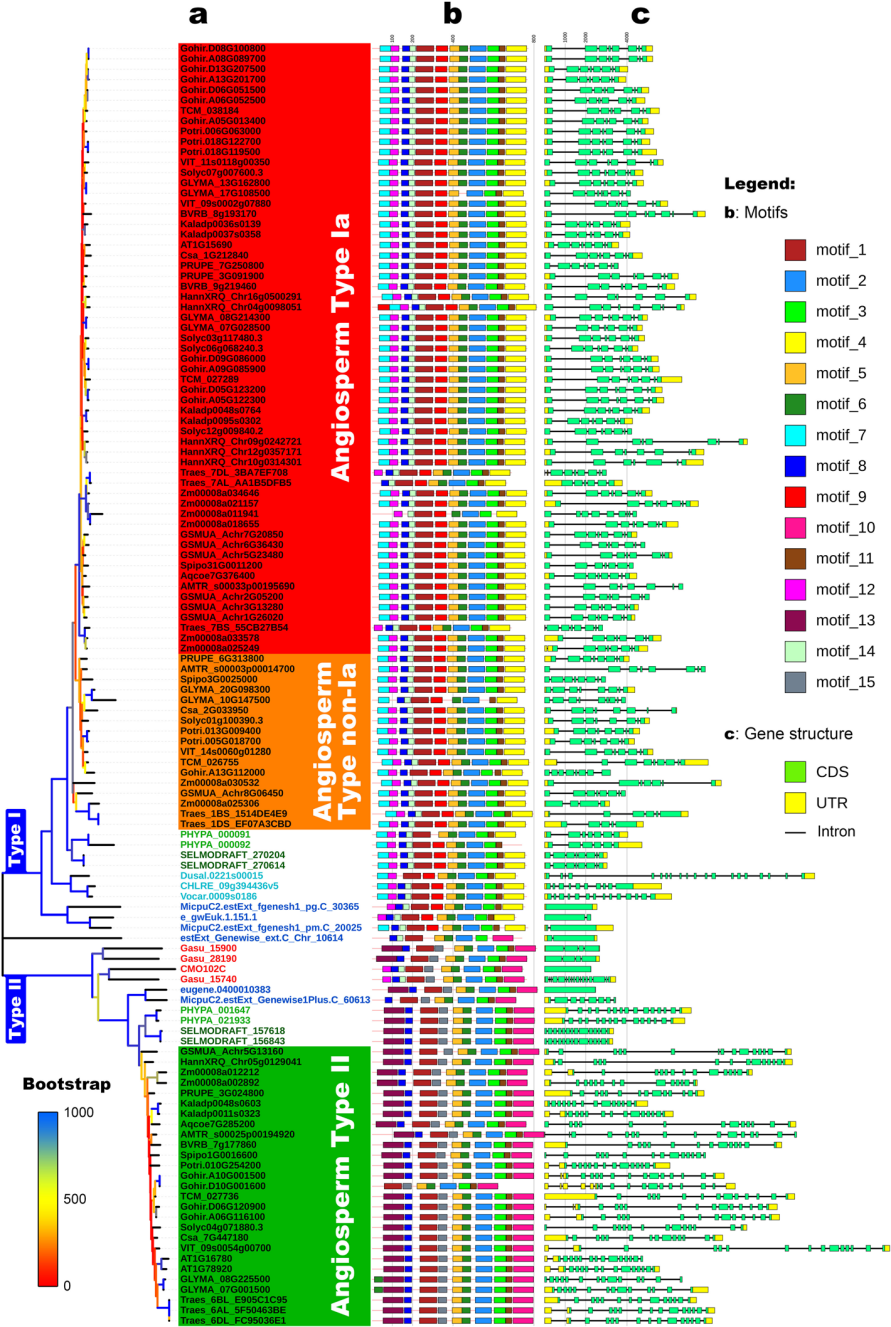
Phylogenetic evolutionary tree, protein motifs, and gene structures of H^+^-PPase gene family members. **a** A neighbor-joining (NJ) phylogenetic tree was constructed using the full-length sequence alignments of 124 H^+^-PPase genes identified using MUSCLE in MEGAX. Bootstrap supports are indicated by the color of the branches. OTUs are labeled as follows: red algae (red); Mamiellophyceae (dark blue); Chlorophyceae (light blue); Bryophytes (light green); Ferns (dark green); Angiosperm (black). Color blocks denote subtypes in angiosperms, with type Ia (red), type non-Ia (orange), and type II (green) denoted. **b** Motifs of the H^+^-PPase proteins. The rectangles indicate the length and positions of motifs. The different colors indicate 15 motifs (left panel). The sequence logo for each motif is shown in Additional file 3. **c** Gene structures of the H^+^-PPase genes. The lengths of rectangles and lines are scaled according to mRNA length. CDSs (green rectangles), UTRs (yellow rectangles), and introns (black line) are denoted

To map the phylogenetic relationships between 124 H^+^-PPase gene family members, two multiple alignment methods (ClustalW [20], MUSCLE [21, 22]) and three phylogenetic inference methods (neighbor-joining, NJ; maximum likelihood, ML; minimum evolution, ME) were employed. In addition, the H^+^-PPase domain sequence and the full-length sequence were also analyzed separately. All resulting phylogenetic trees had similar topologies (Additional file 2). Considering the calculation time, the bootstrap value, and the subsequent analysis needs, the MUSCLE aligned full-length sequence and the NJ method were selected for further analysis. Among the plant H^+^-PPase gene family members identified in the present study (Fig. 1a), only estExt_Genewise_ext.C_Chr_10614 in *Ostreococcus lucimarinus* was on an independent evolutionary branch. The other 123 members of the H^+^-PPase gene family belonged to type I or type II branches. The type I H^+^-PPase gene subgroup was the largest, and accounted for 69.4% of the genes observed, while type II genes accounted for the remaining 29.8%. This may be due to the greater demand for type I H^+^-PPase gene expression in plants, which contributed to the accumulation of these gene copies.

In the angiosperm branches of type I and type II genes, a large number of branch nodes had low bootstrap values. This phenomenon may be the result from few overall differences between the members on the related branches (Additional file 2, Fig. 1a). Among them, the type II H^+^-PPase protein members from the same species belonged to closely related branches, while in the type I group, the opposite was true. Among the type I H^+^-PPases, a large cluster from one branch of angiosperm type I genes with a high bootstrap value (the red background area in Fig. 1a) had structural differences with members on other branches, and the protein sequences of the internal members of this branch had very high similarity. In order to study the possible differences among type I H^+^-PPase genes, this group was classified as subtype Ia, and the remaining type I genes were classified as subtype non-Ia.

By comparing the distribution of H^+^-PPase protein subgroups among algae and higher plants, we identified an unbalanced distribution of gene family members in the early stages of plant evolution. Red algae contain only type II H^+^-PPases, while chlorophyceae green algae (*D. salina*, *C. reinhardtii*, and *V. carteri*), which are more closely related to higher plants, contain only type I H^+^-PPases. However, the relatively primitive mamiellophyceae green algae (*Micromonas pusilla*, *O. lucimarinus*), like bryophytes, ferns, and angiosperms, contain both type I and II H^+^-PPase genes (Table 2).

**Table 2:** The plant H^+^-PPase gene family member subsets

Species group	Species	Distribution of subgroups			
		Type I		Type II	Others
Red algae	*Cyanidioschyzon merolae*			1	
	*Galdieria sulphuraria*			3	
Green algae	*Micromonas pusilla*	2		1	
	*Ostreococcus lucimarinus*	1		1	1
	*Dunaliella salina*	1			
	*Chlamydomonas reinhardtii*	1			
	*Volvox carteri*	1			
Bryophytes	*Physcomitrella patens*	2		2	
Ferns	*Selaginella moellendorffii*	2		2	
Angiosperms		Type Ia	Type non-Ia		
	*Amborella trichopoda*	1	1	1	
	*Spirodela polyrhiza*	1	1	1	
	*Musa acuminata*	6	1	1	
	*Triticum aestivum*	3	2	3	
	*Zea mays*	6	2	2	
	*Aquilegia coerulea*	1		1	
	*Beta vulgaris*	2		1	
	*Helianthus annuus*	5		1	
	*Solanum lycopersicum*	4	1	1	
	*Kalanchoe fedtschenkoi*	4		2	
	*Vitis vinifera*	2	1	1	
	*Arabidopsis thaliana*	1		2	
	*Theobroma cacao*	2	1	1	
	*Gossypium hirsutum*	11	1	4	
	*Populus trichocarpa*	3	2	1	
	*Cucumis sativus*	1	1	1	
	*Glycine max*	4	2	2	
	*Prunus persica*	2	1	1	

In addition, we explored the positions of 124 genes in the background tree (Including 323 seed sequences from database Pfam 32.0, Additional file 4). We found that type I and type II genes are located in the branches where eukaryotes are abundant, and only estExt_Genewise_ext.C_Chr_10614 in *O. lucimarinus* was located distantly from the eukaryotes. This indicates that estExt_Genewise_ext.C_Chr_10614 may therefore represent an H^+^-PPase gene other than type I and type II.

### Structural differentiation of the plant H^+^-PPase gene family members

The mRNA sequence length of the H^+^-PPase genes varied from 2246 bp (e_gwEuk.1.151.1 in *O. lucimarinus*) to 16,779 bp (VIT_09s0054g00700 in grapes), and family members within the same cluster had similar genetic structures. Type I H^+^-PPase genes had relatively fewer exons that were nonetheless longer than those in type II H^+^-PPase genes. The full-length mRNA sequences of type II members were longer than those of type I, and their exons were more often interrupted by introns (Fig. 1c). This phenomenon not only confirms that the two types of members have experienced different evolutionary processes, but also may be one of the reasons for the low expression of type II members.

There were also significant differences in the amino acid sequences of the proteins encoded by the plant H^+^-PPase genes, the shortest of which containing 625 amino acids (Gohir.D10G001600 in cotton, with deletion of the first helix), and the longest containing 853 amino acids (AMTR_s00025p00194920 in *Amborella trichopoda*), with an average of 770 amino acids. We observed an average of 762 residues for type I proteins and 793 residues for type II (including Gohir.D10G001600). The average isoelectric point of type I proteins was 5.33 and that of type II was slightly higher at 5.71 (Additional file 1).

Protein sequence analysis revealed that all H^+^-PPase proteins shared motifs, including motif 1 located at core TM5–TM6, motif 2 located at core TM11–TM12, motif 3 located at TM13, and motif 6 located at TM9–TM10 (Fig. 1b). The K^+^ ion-dependent determinant “GNxxAAIG” motif is located within motif 2 (Additional file 3). The difference between H^+^-PPase type I and type II proteins was mainly reflected in the TM1 helix position of the N-terminus, the motifs 9 (type I) / 15 (type II) in the TM7–TM8 region of the middle section, and the motif 4 (type I) / motif 10 (type II) in TM15–TM16 of the C-terminus (Fig. 1b).

By comparing the distribution of motifs and three-dimensional models (SWISS-MODEL [23]), we found that the structure of gene estExt_Genewise_ext.C_Chr_10614 seems to be similar to that of type II genes (Fig. 1b, Additional file 5). Confoundingly, this gene also has a K^+^ ion-dependent determining domain “GNTTAATG”, which is similar to that of type I members (Additional file 1). These characteristics further confirm the uniqueness of gene estExt_Genewise_ext.C_Chr_10614 in *O. lucimarinus*.

### Duplication events in H^+^-PPase genes in plants

After analyzing 27 plant species, only one pair of tandem repeats was found in moss (PHYPA_000091, PHYPA_000092; Table 1, genes highlighted by bold typeface), and no tandem repeats were identified in angiosperms with frequent duplication events. We searched the Plant Genome Duplication Database (PGDD, http://chibba.agtec.uga.edu/duplication/) for species with 7 or more copies of H^+^-PPase genes (i.e., corn, wheat, soybean, banana, and upland cotton). Although the genomic segmental duplication information of some species (wheat and upland cotton) has not been recorded in the PGDD database, eight pairs of segmental duplications were found in corn, banana, and soybean, two of which were type II H^+^-PPase genes, while the other genes were of the Ia subtype. The number of non-Ia subtype members is small, which may indicate that no fragments containing non-Ia subtype members underwent segmental duplication. The estimated separation time based on the effective synonymous substitution rate (Ks) value of fragment repetition was similar to the WGD date of the species (Table 3).

**Table 3:** Estimated dates for segmental duplication events of H^+^-PPase gene family members in the plant genome duplication database

Species (λ)	Gene pairs		Ks (Mean ± s.d.)	Estimated time (Mya)	WGD (Mya)
*Zea mays* (6.5 × 10^− 9^) [24]	Zm00008a033578	Zm00008a025249	0.4167 ± 0.4910	*	~ 1 2 [25], 70~9 0 [26], ~ 13 0 [27]
	**Zm00008a012212**	**Zm00008a002892**	0.23	17.6923	
	Zm00008a021157	Zm00008a018655	0.27 ± 0.1697	*	
*Musa acuminata* (4.5 × 10^− 9^) [28]	GSMUA_Achr6G36430	GSMUA_Achr7G20850	0.52 ± 0.0743	57.7778	~ 6 1 [28], ~ 6 5 [29], 70~9 0 [26], ~ 13 0 [27]
	GSMUA_Achr1G26020	GSMUA_Achr3G13280	0.5157 ± 0.1242	*	
*Glycine max* (6 × 10^−9^) [30]	GLYMA_07G028500	GLYMA _08G214300	0.1413 ± 0.0847	11.775	5~13 and ~ 12 5 [31–35]
	GLYMA _08G214300	GLYMA _13G162800	1.5033 ± 0.2768	125.275	
	**GLYMA_07G001500**	**GLYMA _08G225500**	0.1625 ± 0.1864	*	

Key: *, Excessive standard deviation, not suitable for estimation. The gene name highlighted by the bold typeface indicates that the gene is a type II member; the remaining genes are of subtype Ia

**Fig. 2:**
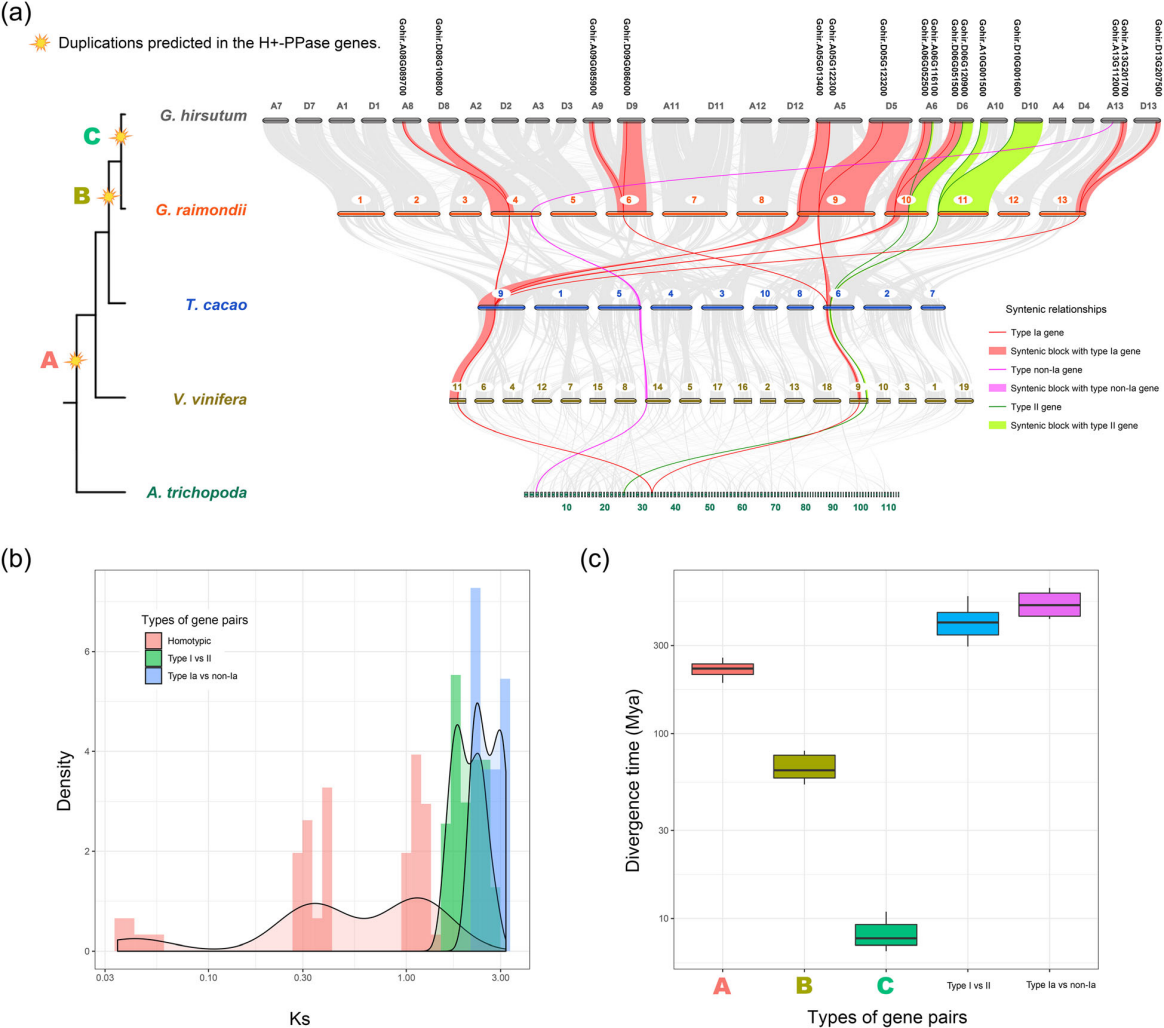
Duplication events of H^+^-PPase genes in *Gossypium hirsutum.* **a** Syntenic relationships of H^+^-PPase genes among *Amborella trichopoda*, *Vitis vinifera*, *Theobroma cacao*, *Gossypium raimondii* and *Gossypium hirsutum*. **b** Distribution of Ks values among H^+^-PPase genes in *Gossypium hirsutum*. **c** Predicted divergence time of H^+^-PPase gene pairs

To further study the duplication events in the evolutionary history of the H^+^-PPase gene family, we used upland cotton as a model because it has the largest number of members from this gene family. According to species evolutionary relationships [19], we analyzed the genome collinearity among a primitive angiosperm (*A. trichopoda*), grape, cocoa, and a diploid cotton (*Gossypium raimondii*), and found that the number of H^+^-PPase genes increased following WGDs in these species, including three amplifications in branches A, B, and C (Fig. 2a). In upland cotton, the distribution of Ks values among different gene pairs for all H^+^-PPases formed four clusters. The first three clusters included the homomorphic gene pairs, while the fourth cluster was composed of heterogeneous H^+^-PPase gene family members (Fig. 2b; Additional file 6). The Ks values between type Ia and non-Ia subtypes and between types I and II in the fourth cluster indicate a highly similar Ks distribution (Fig. 2b). The segregation period of H^+^-PPase members in upland cotton was estimated according to an average synonymous replacement rate of 2.6 bases per 10^9^ years (λ = 2.6 × 10^9^) [36] (Fig. 2c). This calculation indicated that the divergence events among homotypic members were similar to the predicted doubling times caused by three events that occurred in upland cotton as follows: A, the WGD shared by angiosperms (γ event) [37, 38]; B, the WGD in the genus *Gossypium,* which occurred 57–70 Mya [30]; and C, the segregation of ancestors from chromosomes A and D in tetraploid cotton, which occurred between 5 and 10 Mya [36]. Regardless of the differences between type I and type II or between Ia and non-Ia subtypes within type I, similar divergence periods were estimated between heterogeneous H^+^-PPase genes, as indicated by their similar Ks distribution. This suggests that the Ks values between the Ia and non-Ia subtypes has reached saturation, and this phenomenon was also observed in species with a large number of H^+^-PPase genes (Additional file 6). Therefore, the Ks value between heterogeneous members can no longer be used to reliably estimate the separation time, indicating that the differentiation between type I and type II and between Ia and non-Ia subtypes within type I occurred at an early evolutionary stage.

In summary, PGDD data and analysis of H^+^-PPase genes in upland cotton suggest that WGDs have played the most important role in the accumulation of H^+^-PPases in higher plant species.

### Expression patterns of H^+^-PPases in plants

**Fig. 3:**
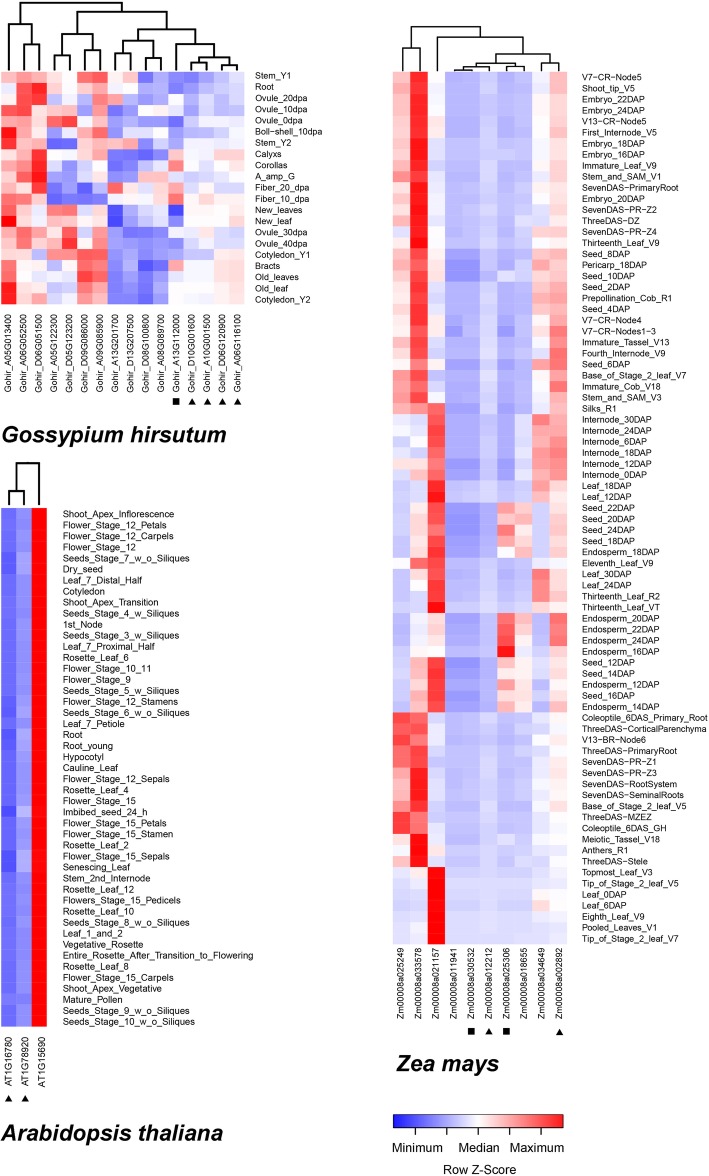
Expression profiles of H^+^-PPase genes in maize, upland cotton, and *A. thaliana.* The level of expression is shown by the color and intensity of each block. The proteins with symbols alongside represent genes of different subtypes as follows: square, type non-Ia; triangles, type II; unlabeled, type Ia. Data source: cotton, GSE70369; corn and *A. thaliana*, online database the Bio-analytic Resource for Plant Biology (BAR) http://bar.utoronto.ca/

To assess the possible functional differentiation among H^+^-PPases in plants, we compared the expression patterns observed in cotton and corn, which have multiple gene family members, and *A. thaliana,* which has fewer members (Fig. 3). In most tissues at different developmental stages, the highest gene expression of H^+^-PPases belonged to the Ia subtype. The expression levels of H^+^-PPases in type II were lower than those in type I, but we observed a smaller difference than what was previously reported [15].

Under typical conditions, compared to *A. thaliana*, the expression patterns in upland cotton and corn were more complex, and each subtype of H^+^-PPase genes had members with very low expression levels. However, most genes were highly expressed in at least one organ or at certain stages of development. Thus, differential expression patterns evolved among plant species with larger number of H^+^-PPase gene family members.

**Fig. 4:**
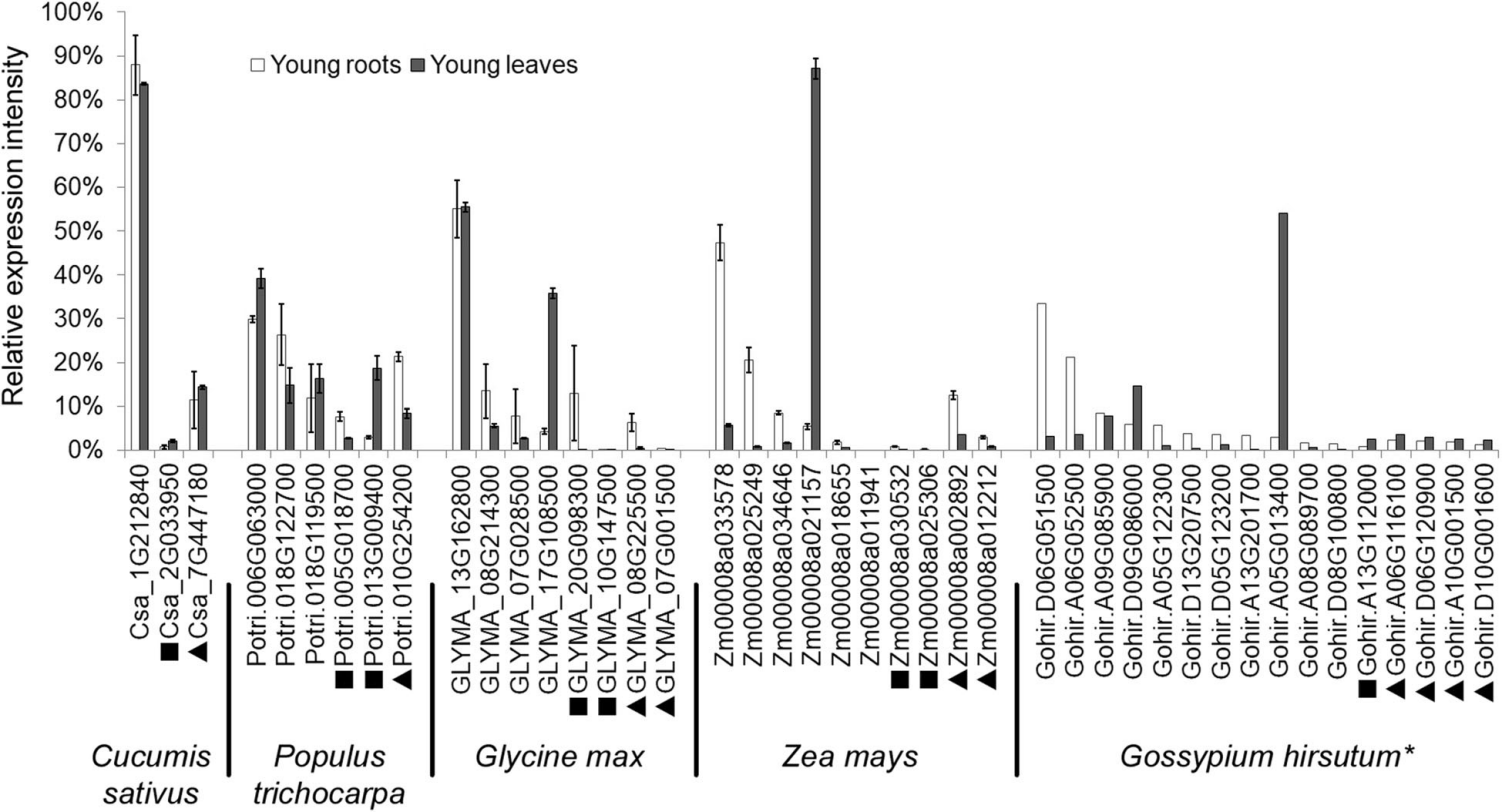
Expression differentiation in H^+^-PPase gene family members. Genes of different subfamilies within one species are arranged in descending order according to expression level in young roots. Black squares indicate type non-Ia; black triangles indicate type II; and unlabeled indicate type Ia. * indicates no biological repeat in GSE70369

The differentiation trends illustrated in Fig. 3 can be more intuitively reflected by comparison of transcriptomes from five species with increasing numbers of gene family members: cucumber (GSM3048829–GSM3048831, GSM1576573–GSM1576580), soybean (GSM1701595–GSM1701597, GSM3714659–GSM3714661), poplar (GSM2565710, GSM2565711, GSM2565718, GSM2565719), maize (PRJNA171684), and upland cotton (GSE70369) (Fig. 4). We found that the expression pattern varied not only among genes of different types or subtypes, but also among copies of the same subtype. Further, the more the number of copies, the more obvious was the extent of differentiation. We speculate that functions of new copies differentiated with unique roles in plant growth and development as the number of copies accumulated.

### Functional divergence in the H^+^-PPases

The DIVERGE v3.0 program [39–41] was used to explore whether amino acid substitutions along different branches of the H^+^-PPase gene family led to the functional bifurcation in the two major branches. Since the estExt_Genewise_ext.C_Chr_10614 in *O. lucimarinus* is on a branch of its own, it was excluded from this analysis.

**Fig. 5:**
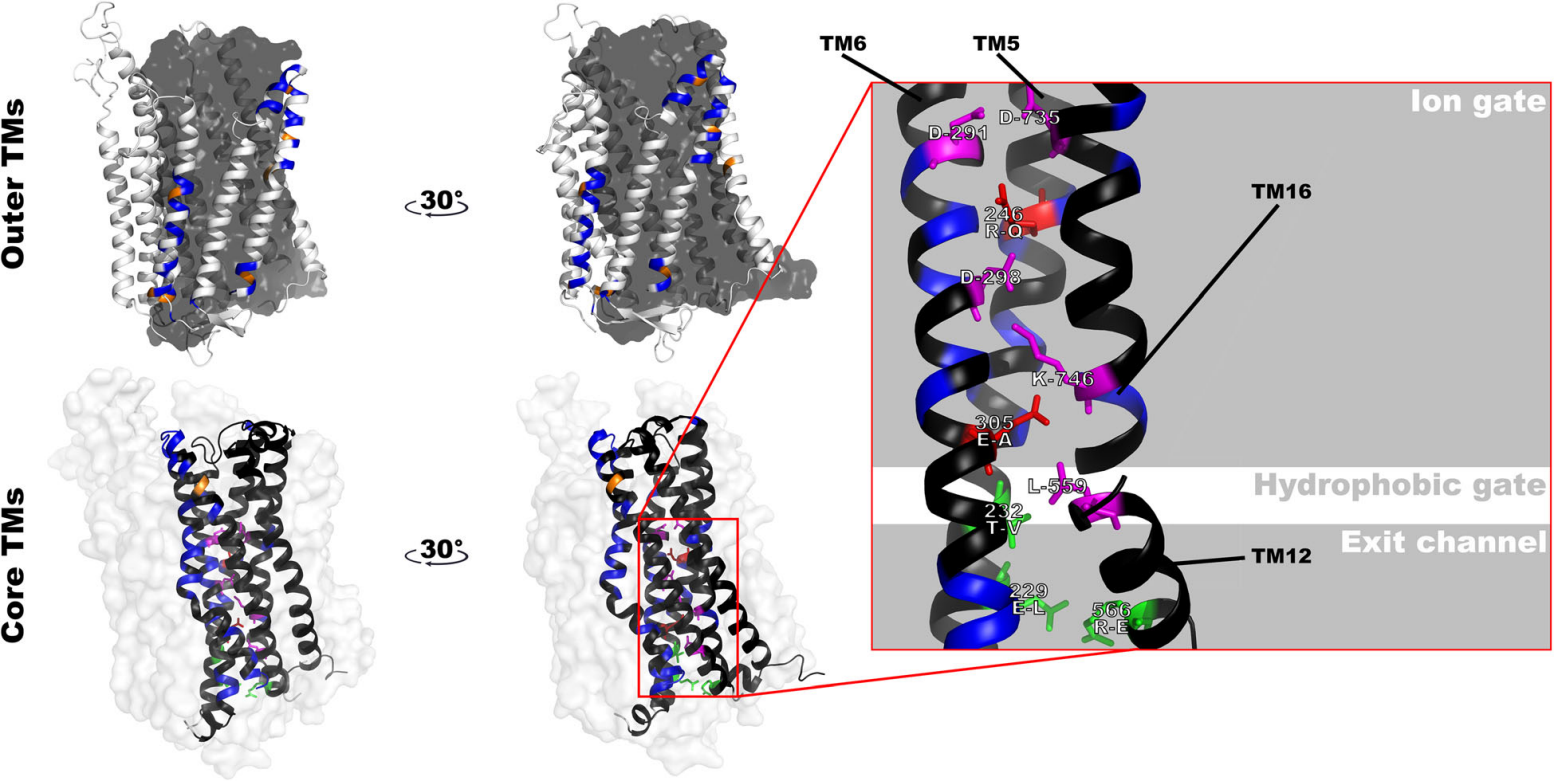
Functional divergence sites in type-I and type-II H^+^-PPases. Left panel: The six core and ten outer transmembrane helices (TM) are shaded in black and white, while sites responsible for both type-I and type-II functional divergence are indicated in orange and blue. Right panel: magnified view of the proton transport pathway. The residues and structures involved in proton transport are labeled as follows (Reference model: 6afu.1.A): two of the 80 Type-II functionally divergent sites (AT1G15690.1 ^R246Q and E305A^) are indicated in red, while other substitutions and conserved residues are indicated in green and magenta respectively. The figure was produced using the pyMOL programs

There was significant functional divergence between type I and type II H^+^-PPases in our dataset, with seven Type-I and 80 Type-II functionally divergent sites identified (Fig. 5; Additional file 7). This indicates that there were different selective constraints on the distribution of amino acid sequences between the two types of H^+^-PPase genes, and that a large number of conserved amino acid sites underwent radical substitution. In type I H^+^-PPases, 10 key amino acid residues were related to diphosphate hydrolysis and proton pump function [16, 17], while five of these were replaced in the type II H^+^-PPases, two of which had undergone Type-II functional divergence (Fig. 5, AT1G15690.1 ^R246Q and E305A^). In terms of the gene structure, significant differences at key sites between these types are likely to cause functional differentiation and limit functional substitution among members. This would also partially explain why all higher plants have both type I and type II H^+^-PPase genes.

### Positive selection in the H^+^-PPase gene family

We next investigated whether there was selective pressure for differentiation among members of the H^+^-PPase gene family on different phylogenetic branches. In the present study, 124 H^+^-PPase genes were analyzed by comparing the “free ratio” model, which assumes that each branch in the phylogenetic tree has different ω values, to the “one ratio” model, which assumes that the whole evolutionary tree has the same ω value. According to the likelihood ratio tests (LRT), the “free-ratio” model was significantly better than the “one-ratio” model, indicating that the different branches of the phylogenetic tree were affected by significantly different selection pressures (Table 4). Using type I and II H^+^-PPase branches as the foreground branches (Additional file 8), “Model A” and “Model A-null” models were compared using the branch site model. This analysis showed that the ω_2_ values of the type I and type II H^+^-PPase branches were significantly higher than 1, and the “Model A” model of the two branches was significantly better than the “Model A-null” model in LRT detection (Table 4). These two major branches of the H^+^-PPase gene family could have been subjected to strong positive selection pressure. We also employed a Bayes Empirical Bayes (BEB) method to identify sites under positive selection with a posterior probability of more than 95%. One site was found amongst the type I genes, while 14 were found among the type II members with a posterior probability of more than 95%, one of which had a posterior probability of > 99% (396 N, AT1G15690.1 ^N284L^) (Table 4, Additional file 9). These results suggest that plant type II H^+^-PPases were subjected to stronger positive selection pressure than type I genes.

**Table 4:** Parameter estimation and likelihood ratio tests for the free-ratio and branch-site models among plant H^+^-PPase genes

Cluster	Model	np ^a^	Ln L	Estimates of parameters (ω_2_)		LRT *P*-value	Positive selected sites ^b^
				Background branch	Foreground branch		
Not required	One-ratio	183	− 135,266.4315	–	–	0.000000000*	Not Allowed
	Free-ratio	363	−134,256.0879	–	–		Not Allowed
Type I	Model A	186	−132,447.573101	0.03780	999.00000	0.000000002*	1
	Model A-null	185	−132,465.522435	1	–		Not Allowed
Type II	Model A	186	−132,461.505672	0.03773	26.50916	0.005794019*	14
	Model A-null	185	−132,465.312309	1	–		Not Allowed
Type Ia ^c^	Model A	118	−66,571.582278	0.03457	1.00000	0.000000000*	None
	Model A-null	117	−67,324.741260	1	–		Not Allowed

Note: *, *p* < 0.01

^a^Number of parameters in the ω distribution

^b^The number of positive-selection sites inferred at posterior probabilities > 95%

^c^Phylogenetic relationships used for the branch-site model analysis (Additional file 10)

In the interior of the angiosperm type I H^+^-PPase branch, we conducted branch site model analysis on the Ia subtype as the foreground branch (Additional file 10). We found that although “Model A” was better than “Model A-null”, the ω_2_ value of the Ia subtype branch was not higher than 1, suggesting that the branch was not positively selected for in the angiosperm type I H^+^-PPases. In addition, five positively selected sites were identified in the branches of the Ia subtype; however, the posterior probability was less than 95% (Table 4, Additional file 9), indicating that the conservation of Ia subtypes was stronger than that of the other plant type I branches.

### Key structural sites in plant H^+^-PPase proteins

**Fig. 6:**
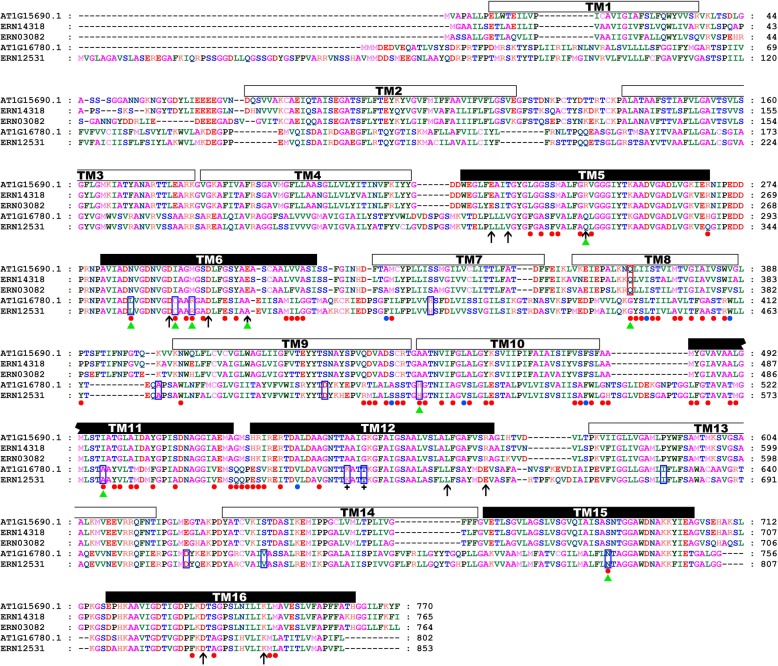
Multiple sequence alignment of H^+^-PPase protein sequences. AT1G15690.1, ERN14318, and ERN03082 belong to type I, among which ERN03082 is non-Ia and the rest are Ia. AT1G16780.1 and ERN12531 belong to type II. Transmembrane helices (TM) in the reference sequence (AT1G15690.1) are outlined and numbered, and the six core and ten outer TMs are indicated in black and white, respectively. Arrows indicate key sites in the reference sequence involved in proton transport. Dots indicate the amino acids responsible for functional divergence (Type-I: blue, Type-II: red). The red and blue outlined boxes indicate amino acids that might be responsible for positive selection of type I and type II H^+^-PPases, respectively. Functional disproportionation sites that are responsible for positive selection or involved in proton transport are indicated with green triangles. Black crosses represent key sites for K^+^ demand

In order to further describe the structural characteristics of plant H^+^-PPases and screen for key sites, a multiple sequence alignment was performed (Fig. 6) with five H^+^-PPase protein sequences from *A. thaliana* and the most primitive angiosperm, *A. trichopoda*.

We found that, of the 80 Type-II functional disproportionation sites, two were involved in proton pump function (Fig. 5, residues indicated in red, AT1G15690.1 ^R246Q and E305A^), and seven sites were responsible for positive selection (one from type I H^+^-PPases, AT1G15690.1 ^Q368G^; six from type II H^+^-PPases, AT1G15690.1 ^N284L, I292C, M295R, A448H, I497A and S688N^). In addition, these nine key sites (Fig. 6, amino acids highlighted by green triangle) are mostly located in the core functional helix (7/9), so amino acid substitutions at these sites may have a significant impact on the function of the proton pump. On the other hand, the key residues that regulate the K^+^ requirement of H^+^-PPase protein are exactly at the positive selection sites for type II H^+^-PPase (Fig. 6, amino acids highlighted by black crosses, AT1G15690.1 ^A541K, G544T^). We therefore hypothesize that the ability to function independently of K^+^ is an evolutionary advantage for the type II members. Using a newly published model [16, 17], we found the key region that defines K^+^ demand (GNxxAAIG) is located in TM12, which is slightly different from previous studies [4, 5].

In summary, among different types, many key regions of H^+^-PPase, including proton transport and potassium ion-dependent determinants, are involved in functional divergence and positive selection to varying degrees. Therefore, different types of H^+^-PPase may play distinct roles.

## Discussion

### Evolutionary processes of H^+^-PPase genes in plants

Although the atypical gene, estExt_Genewise_ext.C_Chr_10614 from *O. lucimarinus*, is an isolated observation in our analysis, it is effectively expressed in transcriptome data (GSM1134625) and can be mutually confirmed with previous results [42]. Therefore, we believe that the plant H^+^-PPase gene family contains at least three different types of protein that originated from the LUCA. Genetic segregation into these types occurred very early in evolutionary history, and each type has experienced a long period of independent evolution. This ancient genetic divergence is similar to that of the V-ATPases and their sibling homologous F-ATPases [2].

In the present study, H^+^-PPase gene family members have diversified ways of presentation in different species, such as: only type I in green algae of class Chlorophyceae; only type II in red algae; type I & type II in higher plants and some green algae. In addition, other type of H^+^-PPase genes have still been found in plants such as “estExt_Genewise_ext.C_Chr_10614”. The genome assembly of the species involved in this study is reliable; however, the reference genome of any species could not be perfect, which also makes it unavoidable to eliminate the possibility of omission in the search results. Therefore, we are still not sure whether this gene family has undergone evolutionary events, such as horizontal gene transfer (HGT) and gene loss, as these events are not uncommon in the early stages of evolution [43].

### Differentiation of angiosperm type I H^+^-PPase subtypes

In angiosperms, type I H^+^-PPase members may have undergone unique differentiation events. In the present study, angiosperm type I H^+^-PPases were divided into Ia and non-Ia subtypes. All angiosperms contained Ia subtype members, and 72% (13/18) of angiosperms had members from the non-Ia subtype. In the most primitive angiosperm- *Amborella*, only two type I H^+^-PPase genes are present, belonging to the Ia and non-Ia subtypes (Table 2). In species that express more than seven H^+^-PPase members, such as upland cotton, the Ks values between the Ia and non-Ia subtypes were significantly higher than those in homotypic members, and reached saturation (Fig. 2b, c, and Additional file 6). There may have been different subtypes of the type I H^+^-PPase genes in the angiosperm ancestor, which gradually evolved to form the structural trunk made up of the present subtypes.

We also found that Ia subtype members had the highest sequence conservation, the highest copy numbers, and were distributed across all 18 angiosperms included in the present study (Table 2). Further, the members of this subtype had the most variable expression patterns, and the members with the highest expression levels were also from this subtype (Figs. 3 and 4). Based on these results, we hypothesize that the Ia subtype could be the dominant H^+^-PPase variant in angiosperms.

### Two evolutionary trajectories of H^+^-PPase gene family

**Fig. 7:**
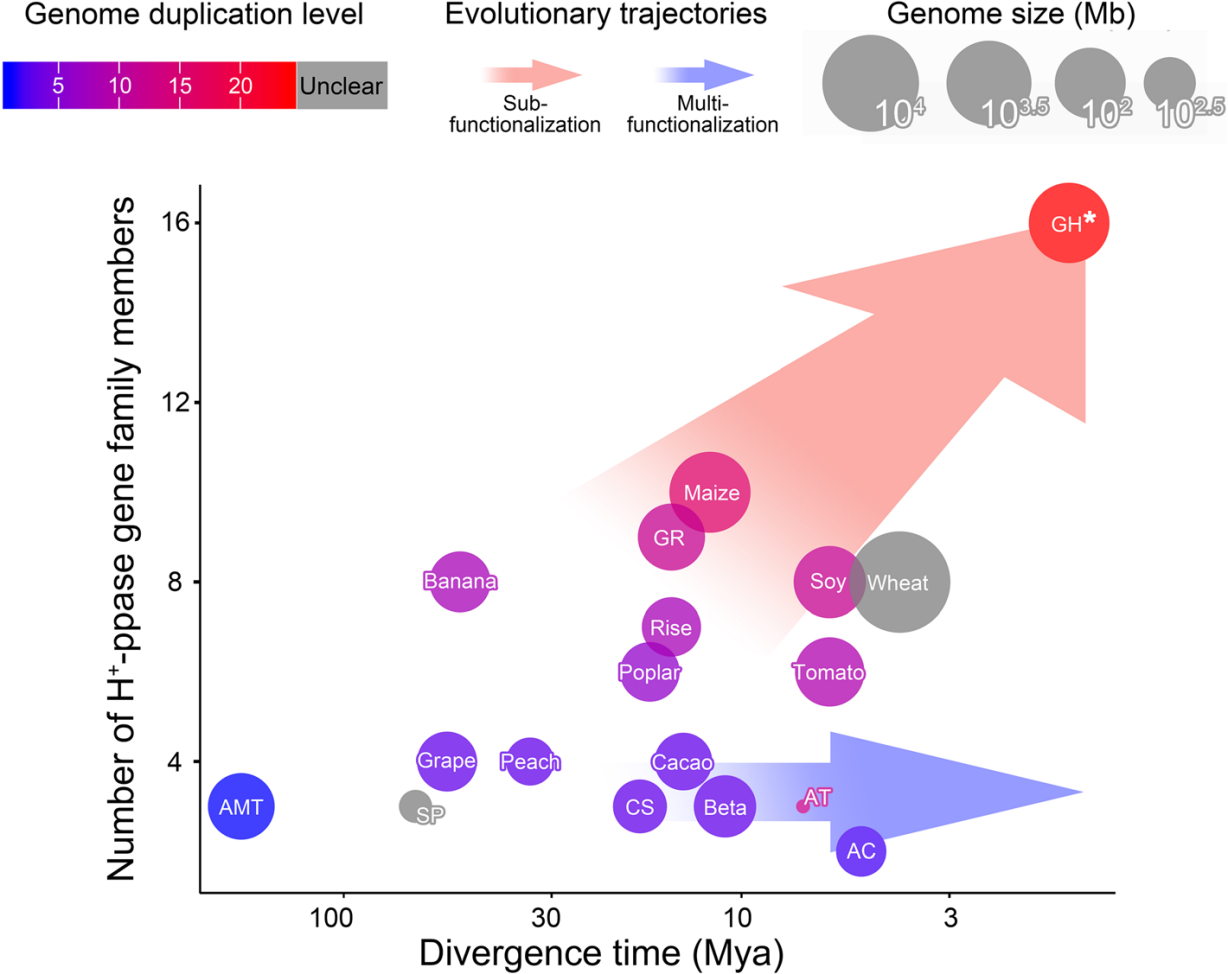
Different evolutionary trajectories of H^+^-PPase gene family members in angiosperms. Genome duplication levels were estimated by reported genome doubling events, with data collated from the online database CoGepedia (http://genomeevolution.org). Divergence time indicates the estimated divergence time of the genus that each species belongs to according to data obtained from the online database Timetree (http://www.timetree.org). *, the “divergence time” of *Gossypium hirsutum* (GH) indicates the time of A- and D-genomes were reunited. AMT: *Amborella trichopoda*; AC: *Aquilegia coerulea*; AT: *Arabidopsis thaliana*; Beta: *Beta vulgaris*; CS: *Cucumis sativus*; Soy: *Glycine max*; GR: *Gossypium raimondii*; GH: *Gossypium hirsutum*; Banana: *Musa acuminata*; Poplar: *Populus trichocarpa*; Peach: *Prunus persica*; Rise: *Oryza sativa*; Tomato: *Solanum lycopersicum; SP: spirodela polyrhiza*; Cacao: *Theobroma cacao*; Wheat: *Triticum aestivum*; Grape: *Vitis vinifera*; Maize: *Zea mays*

Among angiosperms, species with multiple H^+^-PPase genes and those with fewer than four H^+^-PPase genes follow different evolutionary trajectories. Over time, the new species separated from their ancestors and gradually formed two trails. One trail could be characterized by new species evolved accompanying copy number inclement of H^+^-PPase gene (Fig. 7, light red arrow). Species on this trail (e.g., upland cotton) often experienced multiple WGD events (i.e., high numbers of duplicate genes), with multiple H^+^-PPase genes specifically expressed in different developmental stages and tissues. However, the differentiation of expression patterns was mainly concentrated among homomorphic gene types with similar sequences. The differentiation of expression patterns was more obvious as the number of gene family members in a species increased (Figs. 3 and 4). This describes an evolutionary trajectory in which genes have almost the same sequences but exhibit spatiotemporal differentiation, defined as the sub-functionalization.

In contrast, on the other trail, the copy numbers of H^+^-PPase genes in newly emerging species were low (Fig. 7, light blue arrow). Cucumber and *A. thaliana* can be found on this trail, and each of them have no more than three H^+^-PPase genes with stable relative expression levels in different tissues and developmental stages (Figs. 3 and 4). We describe this trajectory of responding to multiple transcriptional needs with one gene as multi-functionalization.

Sub- and multi-functionalization have distinct characteristics during the evolution of a gene family. For example, sub-functionalization avoids the risk of mutation, while multi-functionalization carries a smaller genetic burden.

## Conclusion

Among the 27 plant species examined in the present study, all possessed H^+^-PPase genes, with 124 different H^+^-PPase gene family members identified. The vast majority of these could be divided into two categories: type I and type II, with type I further differentiated into subtypes Ia and non-Ia. There were significant differences in the copy numbers of H^+^-PPase genes among different plant species, and the species with higher copy numbers were usually angiosperms. We also found that the accumulation of H^+^-PPase gene copies in angiosperms was mainly due to WGD events in each species. In lower plants (e.g., red algae and green algae), the different types of H^+^-PPase genes were unevenly distributed, while all higher plants (e.g., vascular plants) contained combinations of both type I and type II H^+^-PPase genes. Phylogenetic analysis, motif analysis, and the prediction of tertiary structures of different H^+^-PPase proteins indicated that “estExt_Genewise_ext.C_Chr_10614” in *O. lucimarinus* is distinct from both type I and type II. We also confirmed significant differences in the expression patterns between type I and type II H^+^-PPase genes, and identified different expression patterns between homomorphic H^+^-PPase genes in species with multiple gene copies. We estimated the functional divergence between type I and type II H^+^-PPase proteins caused by amino acid substitution, and found that two of the ten functionally related key amino acid sites were related to Type-II functional divergence. We also found that both type I and type II H^+^-PPase branches were subjected to very strong positive selection pressures. However, there was no obvious positive selection among members of the Ia subtype in angiosperms. These results improve our understanding of the structural evolution and functional differentiation of the plant H^+^-PPase gene family, and provide a foundation for further exploration of the function and potential applications of this gene family.

Based on significant differences in the number of H^+^-PPase genes in angiosperms and the differentiation among homomorphic members, we propose two gene family evolutionary trajectories (sub- and multi-functionalization) that explain the observed evolutionary phenomena.

## Methods

### Data sources

Twenty-seven representative plants with relatively complete annotated genome data were selected as the research subjects from the APG taxonomy [44] and phylogenetic relationships. Taxonomic evolutionary relationships among species were visualized using the Timetree online tool (http://www.timetree.org/) [45, 46] (Additional file 11). The genomic data were downloaded from the Ensembl Plants dataset (https://plants.ensembl.org) and the Plant JGI Database phytozome v12.1 (https://phytozome.jgi.doe.gov/pz/portal.html). For genome version information, see Additional file 12.

A curated seed alignment containing 323 representative H^+^-PPase proteins was downloaded from Pfam 32.0 [47] (http://pfam.xfam.org/). This seed alignment was used as a background to explore the genetic position of the plant H^+^-PPases.

### Identification of H^+^-PPase gene family members

The hidden Markov model (HMM) (pfam number: PF03030) for the characteristic domain of H^+^-PPase proteins was downloaded from the Pfam database (http://pfam.xfam.org) [47]. HMMER v 3.1 [18] was used to search for candidate genes in the whole protein sequence data of each different species. Because the protein domain of the H^+^-PPase gene family is large (650 amino acids), the sequence coverage rate was more than 80%, and the e-value was less than 1 × 10^− 200^. Proteins with domain separation in intervals of no more than 50 amino acids, a sum of sequence coverage of more than 90%, and protein e-values of less than 1 × 10^− 200^ for each section were used as candidates. The longest transcript of each gene was selected as the candidate member and submitted to SMART (Simple Modular Architecture Research Tool: http://smart.embl-heidelberg.de) [48] for verification. These results were used for downstream analysis of the H^+^-PPase gene family.

### Analysis of H^+^-PPase gene and protein structure

The structural information of gene transcripts was extracted from GFF3 (Generic Feature Format Version 3) annotation files, and the protein motif structure was obtained using the online tool MEME [49, 50] (http://meme-suite.org/tools/meme 5.04). The main parameters were as follows: the search motif type was 15, the distribution number of each motif in the sequence was 0 to 1, the size range was 6 to 100, and the *p* value was less than 10^− 5^. The resulting data were compiled and submitted to the online tool iTol [51] (https://itol.embl.de / 4.3.2) to visualize protein structures.

The protein sequences were submitted to the SWISS-MODEL [23] website for tertiary protein structure prediction. Then, the predicted structure maps of different types of H^+^-PPase proteins were exported using PyMOL (http://www.pymol.org) software.

### Establishment of phylogenetic relationships

We analyzed the results of the two multiple alignment methods (ClustalW and MUSCLE) and three phylogenetic inference methods (NJ, ML, and ME) in MEGAX [52] with 1000 bootstrap replicates to choose stable phylogenetic trees.

Because of the need for functional divergence analysis and positive selection analysis, whole protein sequences were used to construct phylogenetic relationships among H^+^-PPase gene family members. For more accurate subgroup division, we also performed phylogenetic analysis of specific functional domains as a reference.

According to the seed alignment presented in the Pfam dataset, MUSCLE aligned functional domain sequences was used to explore the position of the plant H^+^-PPases in the background tree.

### Analysis of family member expansion

We focused on two replication mechanisms for H^+^-PPase gene family members: segmental duplication and tandem duplication [53]. We considered members of the H^+^-PPase gene family that were no more than 10 genes apart as tandem duplications [54]. Segmental duplication was determined by PGDD, and the corresponding repeat fragments and Ks values [55] were obtained. In addition, for upland cotton, analyses of the genomes of evolutionarily related species and a search for segmental duplications over different periods was conducted with MCscanX (v. python) [56] and blast2.7.1 software. The separation time between the corresponding genes was estimated according to the formula T = Ks/2 λ [55]. The Ks values among gene members in different species were calculated with CODEML [57].

### Gene expression pattern analysis

Transcriptome data were mined from the Gene Expression Omnibus DataSets (GEO), the Sequence Read Archive (SRA) database, and the Bio-analytic Resource for Plant Biology (BAR) http://bar.utoronto.ca/. For species whose gene IDs were difficult to match to the ID in the high-throughput data, we used Salmon [58] (v 0.13.1) to analyze the expression levels of all genes in a selected species and extract the transcripts per million (TPM) value for the H^+^-PPase genes, after quality detection and filtering using FastQC (v 0.11.8) and Trimmomatic (v 0.38) [59].

In order to compare the expression of H^+^-PPase genes in specific tissues of different species, we calculated and compared the relative expression.

The following formula was used for calculating relative gene expression intensity:

$$ \mathrm{Relative}\ \mathrm{expression}\ \mathrm{in}\mathrm{tensity}\ \mathrm{of}\ \mathrm{target}\ \mathrm{gene}=\frac{\mathrm{Target}\ \mathrm{gene}\ \mathrm{expression}\ \mathrm{level}\ }{\sum \mathrm{Expression}\ \mathrm{level}\ \mathrm{of}\ \mathrm{each}\ \mathrm{member}\ \mathrm{of}\ \mathrm{the}\ \mathrm{gene}\ \mathrm{family}\ \mathrm{in}\ \mathrm{the}\ \mathrm{sample}}\times 100\% $$

### Functional divergence analysis and positive selection analysis

The DIVERGE v3.0 [39–41] software was used to determine whether amino acid substitutions in the H + -PPase gene family caused significant changes in site-specific differences according to either the evolutionary rate (Type-I) or amino acid properties (Type-II) after the emergence of two paralogs.

Analysis of the positive selection in H^+^-PPase gene family members was based on the CODEML program [57, 60, 61] in PAML, using the ML method and the branch-site model to identify whether a particular evolutionary branch was positively selected for. Subsequently, we searched for positively selected sites on that branch.

## Supplementary information

**Additional file 1.** Details of 124 gene family members and their encoded proteins.

**Additional file 2.** Phylogenetic trees constructed by different methods.

**Additional file 3.** Sequence logo of each motif in Fig. 1.

**Additional file 4.** Genetic positions of 124 members in the background of family’s seed alignment tree.

**Additional file 5.** Protein structures of Plant H ^+^ -PPase protiens.

**Additional file 6.** Ks values in species with a large number of H ^+^ -PPase genes.

**Additional file 7.** Functional divergence between types of the H ^+^ -PPase gene family.

**Additional file 8.** Phylogenetic trees used in positive selection analysis.

**Additional file 9.** Positive selection sites found in analysis.

**Additional file 10.** Phylogenetic trees used in positive selection analysis of type Ia.

**Additional file 11.** Taxonomic relationships among 27 representative plants.

**Additional file 12.** Data sources.

## Data Availability

The ID or other identifying information of the relevant data set supporting the results of this article has been indicated in the place where they appear. The information relevant to the genome data analysed in this study are available in the Additional file 12.

## Abbreviations

AVP: *Arabidopsis* vacuolar proton pyrophosphatases
BEB: Bayes empirical bayes
CDS: Coding sequence
GEO: Gene Expression omnibus
GFF3: Generic feature format version 3
H^+^-PPase: Proton-translocating pyrophosphatase
HMM: Hidden markov model
JGI: Department of energy joint genome institute
Ks: Synonymous base substitution rates
LRT: likelihood ratio test
LUCA: Last universal common ancestor
ME: Minimum evolution
ML: Maximum likelihood
mRNA: Messenger ribonucleic acid
Mya: Million years ago
NCBI: National center for biotechnology information
NJ: Neighbor-joining
OTU: Operational taxonomic unit
PGDD: Plant genome duplication database
PPi: Pyrophosphate
SMART: Simple modular architecture research tool
SRA: Sequence Read Archive
TM: Transmembrane helix
TPM: Transcripts per million
UTR: Untranslated region
V-PPase: Vacuolar proton pyrophosphatases
WGD: Whole-genome duplication
