# Supplementary figures and images for: The structure, functional evolution, and evolutionary trajectories of the H+-PPase gene family in plants
Source: BMC Genomics. 2020 Mar 2;21:195. doi: 10.1186/s12864-020-6604-2 (PMC7053079; doi:10.1186/s12864-020-6604-2)

Additional file 2, phylogenetic trees constructed by different methods.


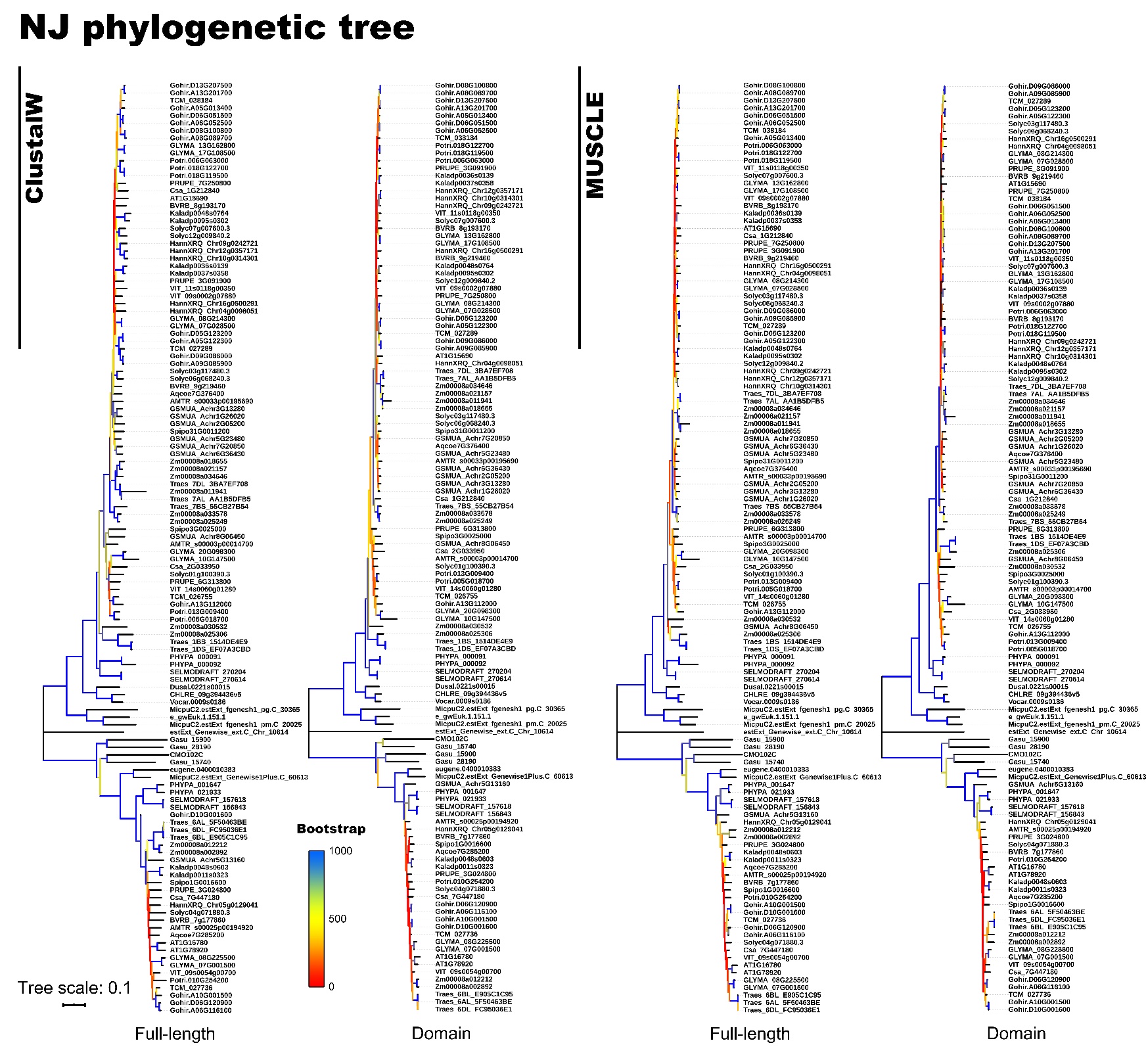


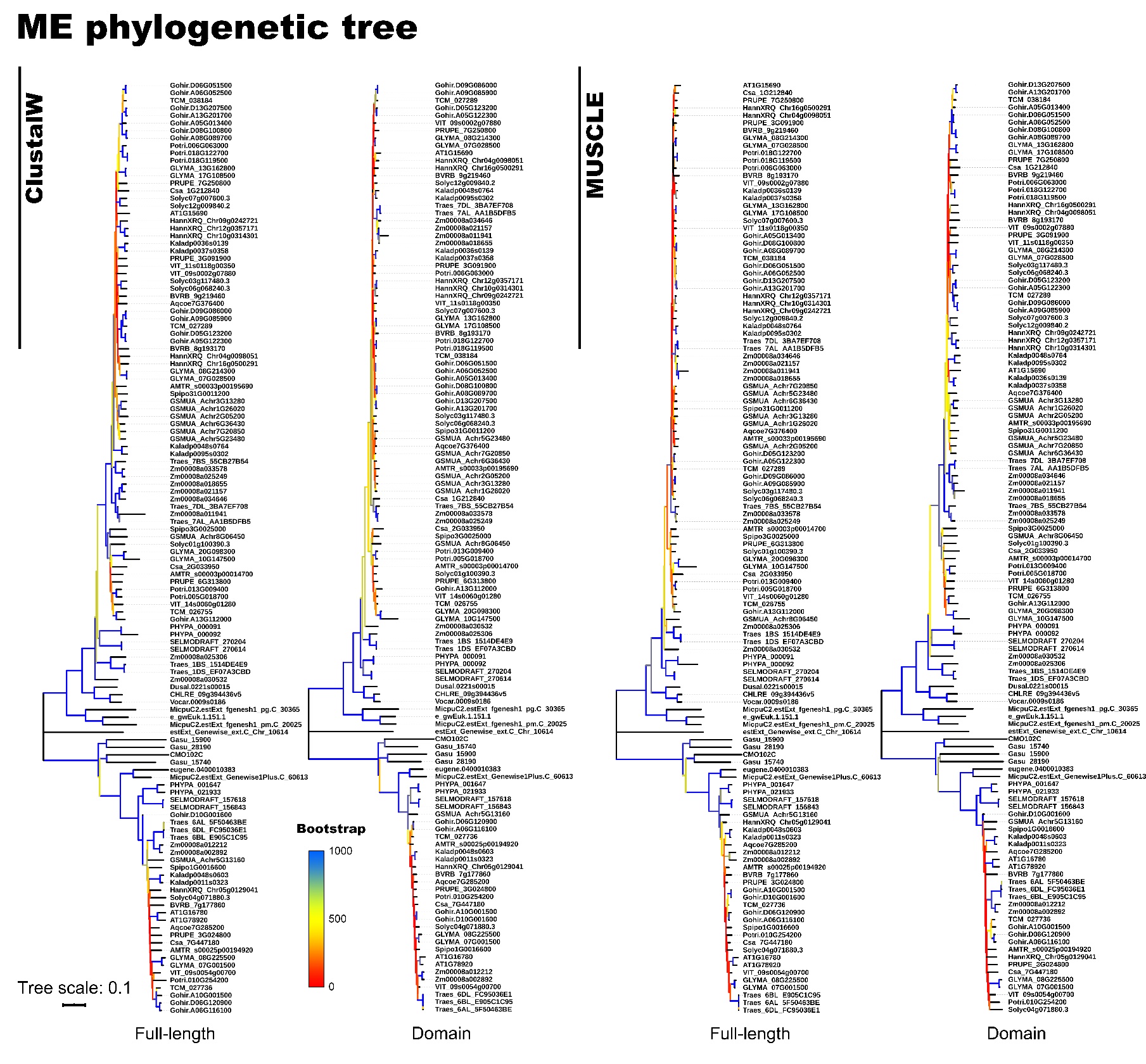


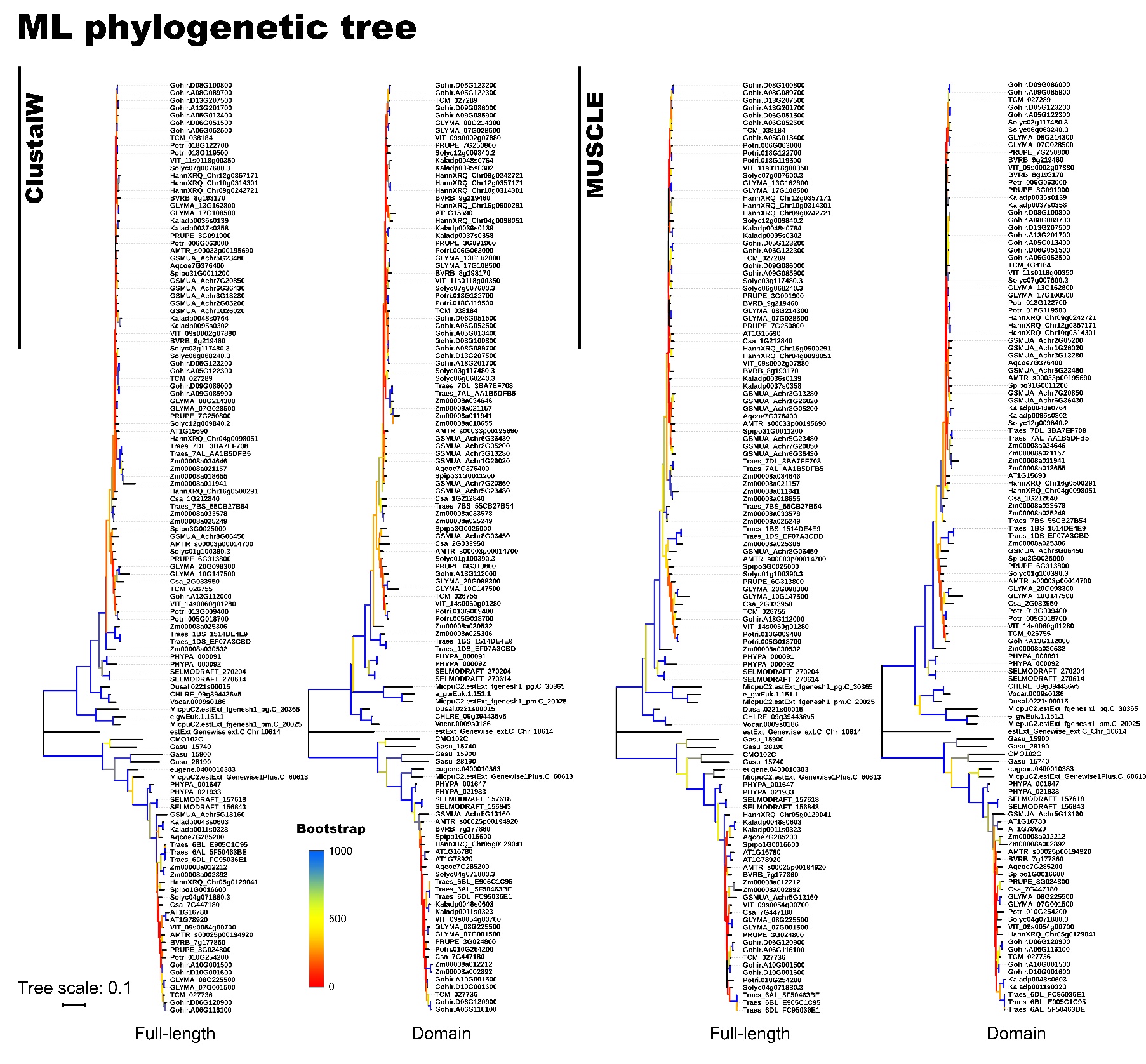

Supplement: Supplementary file 2 — Additional file 2. Phylogenetic trees constructed by different methods. [file 12864_2020_6604_MOESM2_ESM.docx]

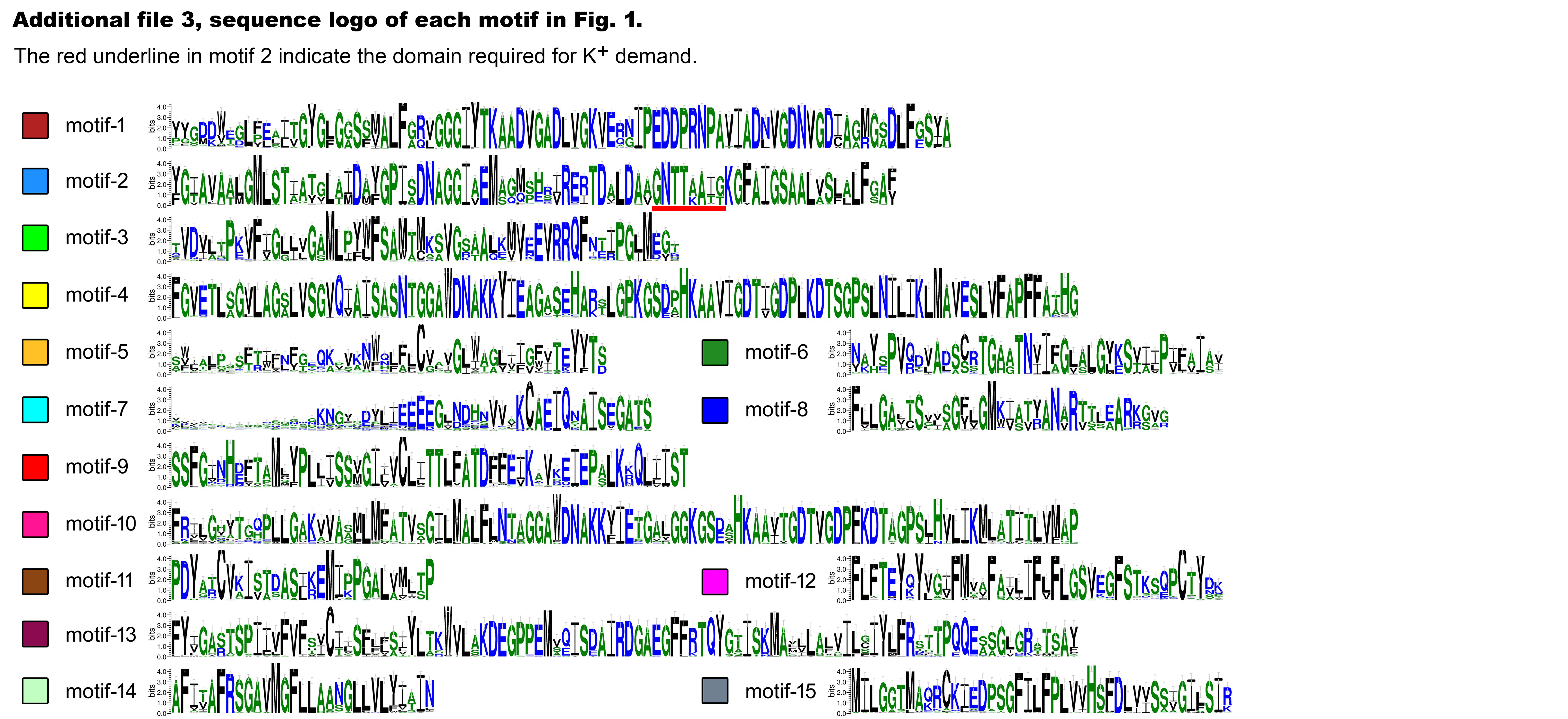

Supplement: Supplementary file 3 — Additional file 3. Sequence logo of each motif in Fig. 1. [file 12864_2020_6604_MOESM3_ESM.jpg]

Additional file
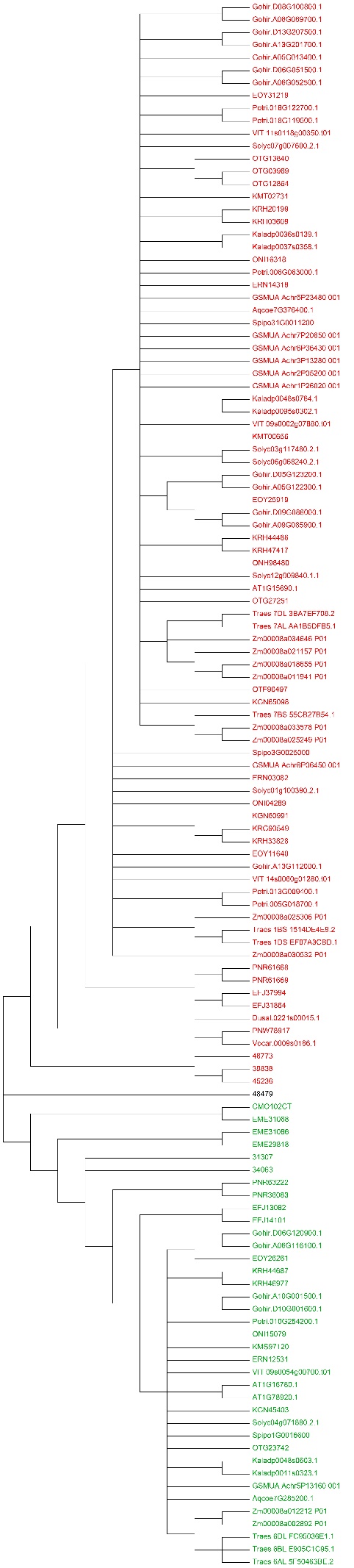
8, phylogenetic trees used in positive selection analysis.

Supplement: Supplementary file 8 — Additional file 8. Phylogenetic trees used in positive selection analysis. [file 12864_2020_6604_MOESM8_ESM.docx]

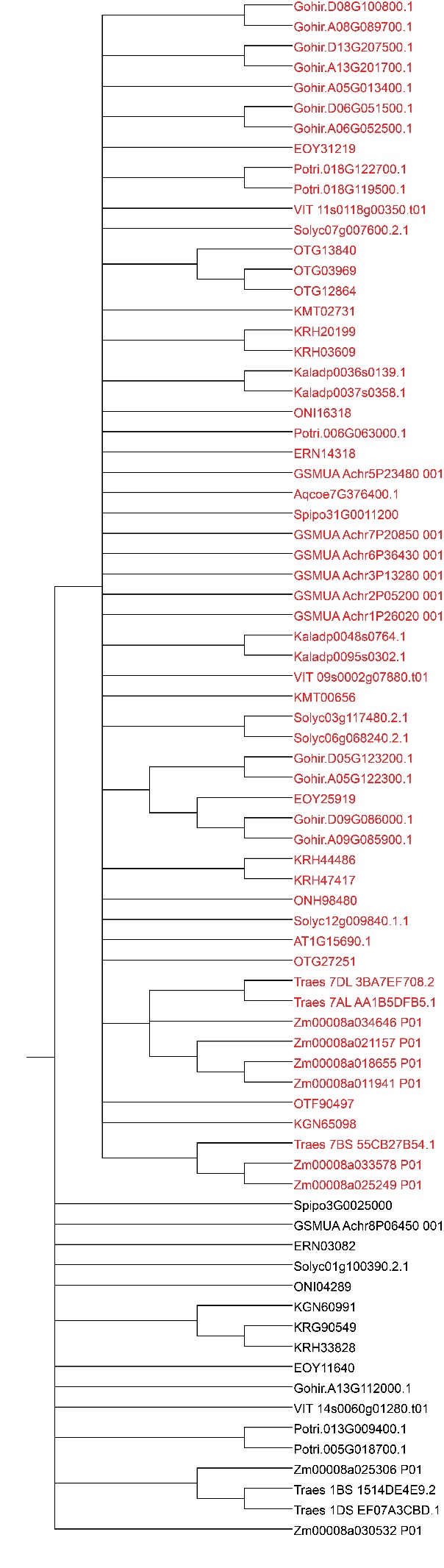
Additional file 10, phylogenetic trees used in positive selection analysis of type Ia.

Supplement: Supplementary file 10 — Additional file 10. Phylogenetic trees used in positive selection analysis of type Ia. [file 12864_2020_6604_MOESM10_ESM.docx]

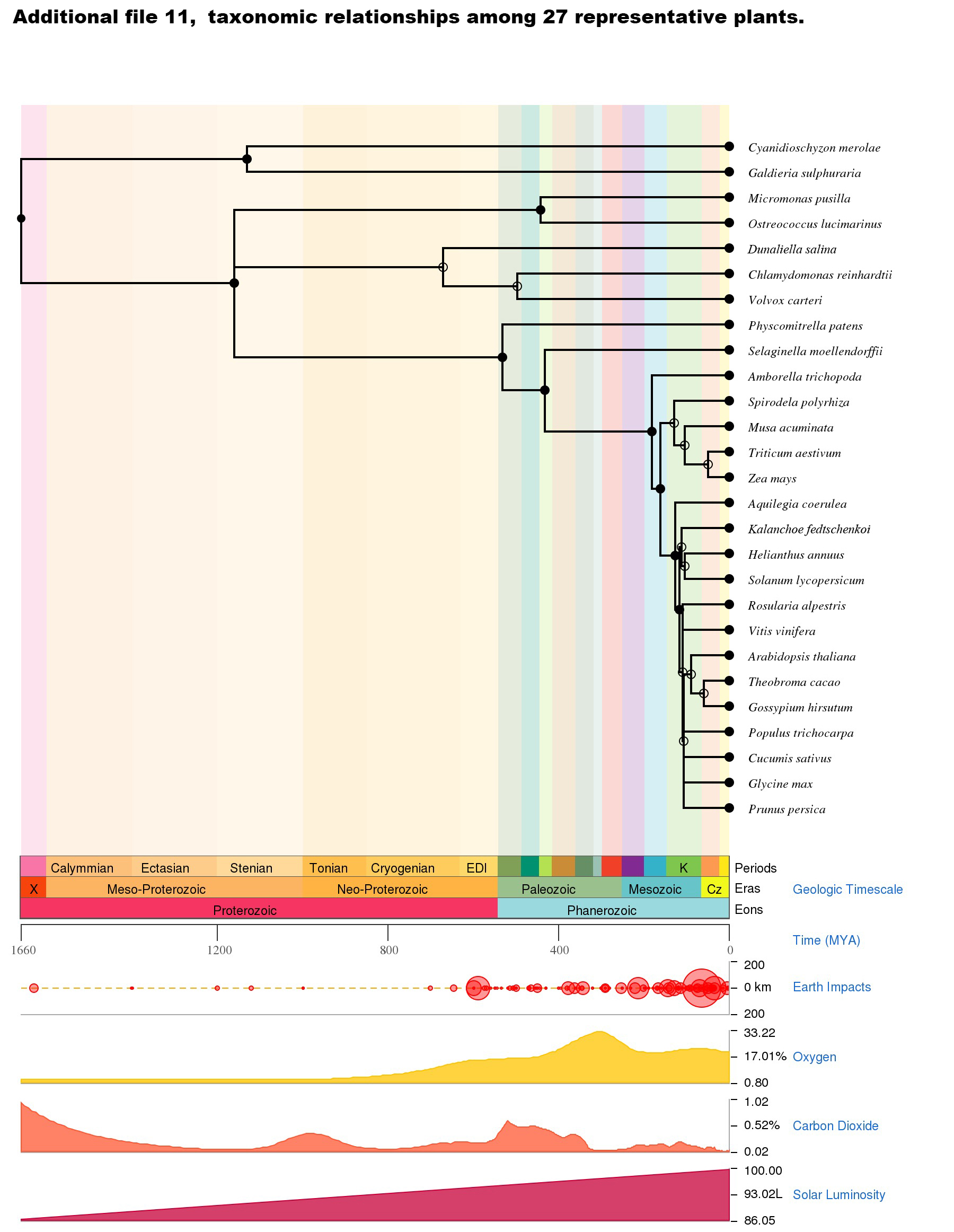

Supplement: Supplementary file 11 — Additional file 11. Taxonomic relationships among 27 representative plants. [file 12864_2020_6604_MOESM11_ESM.jpg]
