# Supplementary material for: The structure, functional evolution, and evolutionary trajectories of the H+-PPase gene family in plants
Source: BMC Genomics. 2020 Mar 2;21:195. doi: 10.1186/s12864-020-6604-2 (PMC7053079; doi:10.1186/s12864-020-6604-2)
Supplement: Supplementary file 4 — Additional file 4. Genetic positions of 124 members in the background of family’s seed alignment tree. [file 12864_2020_6604_MOESM4_ESM.docx]

Additional file 4, genetic positions of 124 members in the background of family's seed alignment tree.


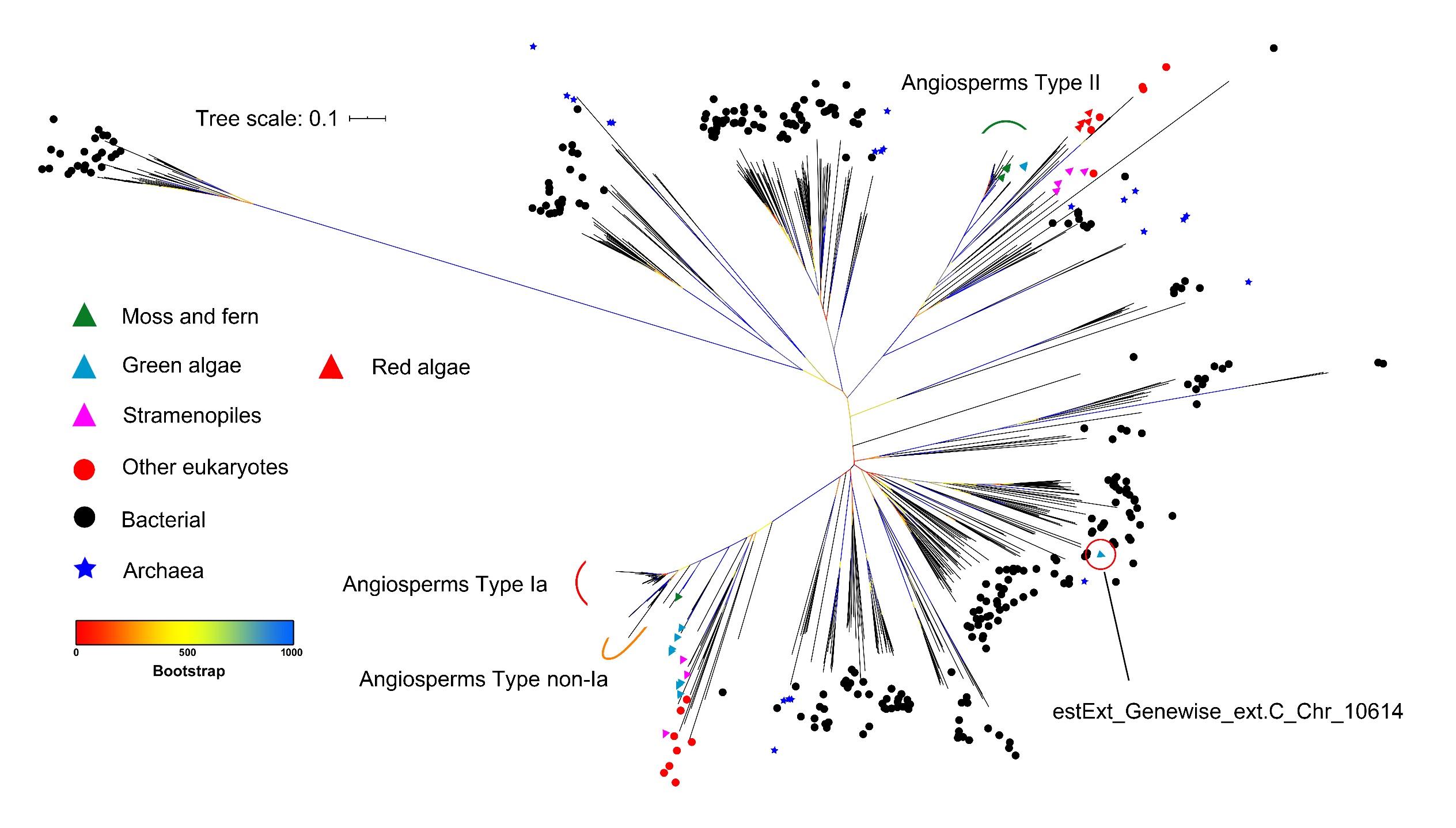


The legend shows the proteins from different species groups. The curves of different colors show the position of subtypes of gene family members in angiosperms. Bootstrap supports are indicated by the color of the branches.
